# Editorial Comment: Dorsolateral fibromuscular tissue preservation during artificial urinary sphincter cuff placement is associated with low infection and erosion rates

**DOI:** 10.1590/S1677-5538.IBJU.2020.01.04

**Published:** 2020-01-13

**Authors:** F Cheung, A Fathollahi, E Vertosick, TR Jarvis, D Katz, JS Sandhu, Luciano A Favorito

**Affiliations:** 1 Memorial Sloan Kettering Cancer Center, New York, NY, USA; 2 New York Medical College, New York, NY, USA; 3 Prince of Wales Clinical School, Randwick, NSW, Australia; 4 Men's Health Melbourne, New York, NY, USA; 1 Unidade de Pesquisa Urogenital - Universidade Estadual do Rio de Janeiro - Uerj, Rio de Janeiro, RJ, Brasil; 2 Serviço de Urologia, Hospital Federal da Lagoa, Rio de Janeiro, RJ, Brasil

## COMMENT

In this important paper Dr. Cheung and cols. from New York - USA, presented a modified technique of artificial urinary sphincter (AUS) placement with dorsolateral fibromuscular tissue of bulbo-spongiosus muscle (BSM) preservation.

The authors proposed a technique that is associated with low rates of erosion and infection in patients with high risk of complications (post radiotherapy, bladder neck contracture, metastatic prostate cancer). BSM, also known as the bulbo-cavernosus muscle, is a paired striated muscle originating from the perineal body and fixed in a median raphe involving the bulbar urethra (1, 2). BSM is related to the expulsion of seminal fluid and urine, especially the last drop, from the bulbar urethra by rhythmic contractions, acting as a pump to evacuate the contents of the bulbar urethra (3). During surgery, the authors performed a dorsal dissection of the urethra outside the BMS and made the inclusion of the dorsolateral fibromuscular tissue of the BMS in the cuff placement.

The authors concluded that the preservation of dorsolateral fibromuscular tissue of BMS during AUS placement is an effective means to achieve a low risk of erosion with good functional outcomes with low rates of re-operation and excellent long-term continence. The study is retrospective but the description of a 74% success index is amazing and this new technique with BSM preservation is very interesting. This article presents a good overview of the surgical treatment of erectile dysfunction-related Peyronie's disease, covering the best indication of the various possible surgical alternatives. To conclude, we also emphasize that pre-operative counseling about the realistic outcomes of surgery in PD is mandatory to achieve adequate postoperative satisfaction rates.
